# *Trichoderma longibrachiatum* (T6) Peptaibols Inhibiting the *Monilia yunnanensis* Growth and Inducing Pear Fruit Resistance in Its Infection

**DOI:** 10.3390/antiox13121517

**Published:** 2024-12-12

**Authors:** Hang Lv, Shuwu Zhang, Nan Ma, Solomon Boamah, Bingliang Xu

**Affiliations:** 1 College of Plant Protection, Gansu Agricultural University, Lanzhou 730070, China; 107332102388@st.gsau.edu.cn (H.L.); 107332102383@st.gsau.edu.cn (N.M.); solomonboamah@st.gsau.edu.cn (S.B.); 2 Biocontrol Engineering Laboratory of Crop Diseases and Pests of Gansu Province, Lanzhou 730070, China; 3 State Key Laboratory of Aridland Crop Science, Gansu Agricultural University, Lanzhou 730070, China

**Keywords:** pear brown rot, *Trichoderma longibrachiatum*, metabolites, peptaibols, biocontrol efficacy

## Abstract

Pear fruit brown rot, caused by *Monilia yunnanensis*, affects pear fruit yields and quality. The present study determined *Trichoderma longibrachiatum* T6 (T6) peptaibols as a biological control alternative to synthetic fungicides and assessed its efficacy against *M. yunnanensis* through dual plate culture and surface spraying at different concentrations. T6 peptaibols effectively inhibited *M. yunnanensis* growth, achieving an 85.99% inhibitory rate at 1250 µg/mL after inoculation on PDA medium for 5 days, and 84.57% control efficacy on pear fruit with the same concentration at 6 days. Treatment with T6 peptaibols significantly decreased the average contents of malondialdehyde (MDA) and hydrogen peroxide (H_2_O_2_), as well as electrolyte leakage, by 31.99%, 27.93%, and 21.00% from days 1 to 9 post-inoculation, respectively, in comparison to the negative control. Additionally, the average antioxidant enzyme activities of catalase (CAT), superoxide dismutase (SOD), peroxidase (POD) and polyphenol oxidase (PPO) increased by 86.27%, 56.76%, 25.94%, and 47.88%, respectively; the average defense enzyme activities of phenylalanine ammonia-lyase (PAL), lipoxygenase (LOX), chitinase (CHI), and β-1,3-glucanase (β-Glu) increased by 63.00%, 55.70%, 26.19%, and 16.34%, respectively. Moreover, the expression levels of the antioxidant and defense-related genes (*CAT*, *SOD*, *POD*, *PPO*, *CHI*, *LOX*, *PAL*, *β-Glu*) were significantly upregulated by 2.80, 2.81, 3.03, 2.79, 3.37, 2.49, 2.73, and 1.83-folds at 3 days after inoculation compared to the negative control. Thus, T6 peptaibols effectively reduced the pathogen infection through growth inhibition and antioxidant defenses, thereby boosting fruit immunity.

## 1. Introduction

Pear is one of the most important fruits produced and consumed around the world, in more than 70 countries and regions, growing on trees and harvested from late summer to mid-autumn [1,2]. China is the world’s leading country in pear production, accounting for 65.70% of world production and 72.30% of world growing area [3]. Over the past decade, there has been a notable increase in both the cultivation area and production of pear by 27.70% and 65.30%, respectively [4]. However, the persistence of pre- and post-harvest diseases is primarily attributed to pathogen infection, with significant losses for pear yields [5].

Pear brown rot is one of the major diseases affecting the pear industry, causing significant yield losses during both pre- and post-harvest stages, and leading to severe economic losses [6,7]. In China, pear brown rot caused by *Monilia yunnanensis* is the most widespread, occurring in all the main regions, including Beijing, Hebei, Gansu, Xinjiang, and Shaanxi [8]. However, the current control of pear brown rot mainly relies on chemical fungicides. With the increasing resistance of pathogenic microorganisms, the effectiveness of chemical fungicides has gradually declined [9]. Therefore, there is an urgent need to develop new alternative methods to manage pear brown rot disease.

Biological control, recognized as an environmentally friendly, efficient, and sustainable disease management strategy, has demonstrated considerable potential in controlling fruit brown rot disease [10]. Numerous studies have shown that varieties of antagonistic microorganisms exhibit significant efficacy in controlling this disease [11,12,13]. Among them, *Trichoderma* spp. stand out as an important group of biocontrol fungi, and have attracted considerable attention, are widely distributed in nature and characterized by rich genetic diversity [14,15]. In recent years, the metabolites of *Trichoderma*, specifically peptaibols, have been found to exhibit broad-spectrum antagonistic activity against various plant pathogens. Studies have shown that peptaibols extracted and purified from *T. reesei* QM9414 effectively inhibit several plant pathogenic filamentous fungi, including *Alternaria alternata* SzMC 16085 and *Fusarium keratoplasticum* SzMC 11414 [16]. Additionally, Marik et al. [17] reported that peptaibols extracts from *T. gamsii* SZMC 1656 and *T. koningiopsis* SZMC 12500 possess significantly inhibitory effects on bacteria and yeasts. However, the effects of peptaibols from *T. longibrachiatum* T6 (T6) on the growth of *M. yunnanensis* have not yet been investigated.

In addition to the directly inhibiting effect of *Trichoderma* on pathogen growth, some studies have demonstrated that *Trichoderma* peptaibols can trigger plant defense responses, thereby significantly enhancing plant resistance to diseases. Trichokonins, a class of antimicrobial peptaibols that has been isolated from *T. pseudokoningii* SMF2, has been shown to induce both defense responses and systemic resistance of tobacco (*Nicotiana tabacum* var. *samsun*) against tobacco mosaic virus (TMV) infection, by increasing the activity of phenylalanine ammonia-lyase (PAL) and peroxidase (POD) and the production of reactive oxygen species (ROS) and phenolic compounds [18]. Moreover, the peptaibol TK VI extracted from *T. pseudokoningii* SMF2 not only promoted moth orchid growth but also enhanced its systemic resistance to gray mold caused by *Botrytis cinerea*. This was achieved by inducing the activity of defense-related enzymes (POD, polyphenol oxidase (PPO) and PAL) and resistance-associated enzymes (superoxide dismutase (SOD) and catalase (CAT)), while reducing the levels of malondialdehyde [19]. However, the mechanism for T6 peptaibols inducing the resistance of pear fruit to *M. yunnanensis* infection remains unclear.

In our previous study, we found that 20-fold dilutions of T6 fermentation presented significant antagonistic activity on the *M. yunnanesis* mycelial growth that caused peach brown rot [20]. However, there is a lack of information concerning how T6 peptaibols inhibit the pear brown rot pathogen in *M. yunnanesis* growth and induce pear resistance to this infection at physiological and molecular levels. Therefore, this study aims to investigate (i) the inhibitory effect of T6 peptaibols on *M. yunnanensis* growth, (ii) the control efficacy of T6 peptaibols on pear brown rot disease and (iii) the induction effect of T6 peptaibols on pear resistance to brown rot disease.

## 2. Materials and Methods

### 2.1. Fungal Inoculum Preparation

The biocontrol strain *T. longibrachiatum* T6 (T6) and pathogenic fungus *M. yunnanesis* were obtained from Gansu Agricultural University’s Laboratory of Plant Pathology and were cultured on potato dextrose agar (PDA) in Petri dishes at 25 °C for 5 days. The fruits of the *Pyrus bretschneideri* Rehd cultivar that was susceptible to brown rot disease were collected from Jingning pear orchard, Gansu Province, China. At commercial maturity (about 120 days after full flowering), healthy pear fruits with uniform size were collected for inoculation in vivo.

### 2.2. Peptaibols from T6 Preparation

The conidia suspension T6 was prepared according to the method described by Zhang et al. [21]. A conidia suspension of 1.0 × 10^6^ conidia per mL was quantified into a YM medium at a rate of 1.5% and placed in a shaking incubator at 28 °C with a shaking speed of 180 rpm for 8 days. After fermentation, the broth was filtered twice through three layers of sterile filter paper under vacuum. The filtrate was then centrifuged at 8000 rpm for 10 mins at 4 °C to collect the supernatant. The supernatant was filtered through a 0.22 μm membrane filter. The filtered fermentation broth was concentrated using a rotary evaporator to obtain crude solids.

The XAD-2 macro-porous resin was first placed in a beaker and soaked in anhydrous ethanol for 2 h to activate the resin. Thereafter, the resin was discarded and the ethanol washed with sterile water to deal with the ethanol smell. Following the washing step, a chromatography column (3 × 50 cm, column volume 350 mL) was prepared and the pre-processed macro-porous resin was then carefully added to the column wall using a glass rod to ensure uniform packing. The resin was packed until the column bed level reached approximately 5 cm from the top. Subsequently, the column was connected to a constant flow pump and flushed with 100% pure water at a flow rate of 1 mL/min for two column volumes. The crude solid of T6 fermentation was filtered through a 0.22 μm membrane and dissolved in 2 mL of methanol. This solution was then slowly introduced to the surface of the resin, ensuring that this was just submerged in the crude solution. The column was then eluted with three elution gradients (100% water, 40:60 water/methanol, and 100% methanol, as outlined in Table 1) at a flow rate of 1 mL/min to obtain fractions with two column volumes (700 mL fractions). The fractions eluted with 100% methanol were collected, and crude peptaibols were subsequently obtained following concentration using a rotary evaporator.

### 2.3. The Inhibitory Effect of T6 Peptaibols on M. yunnanensis and Disease Control

Accurate measurements of 25, 50, 75, 100, and 125 mg of T6 peptaibols were weighed and dissolved in 1 mL of methanol, respectively, and then 1 mL of different concentrations of T6 peptaibols was added to 99 mL of sterilized PDA media, respectively to make the final T6 peptaibols concentrations at 250, 500, 750, 1000, and 1250 μg/mL, while 1 mL of sterile water and 1 mL of methanol were added to 99 mL of sterilized PDA media, serving as the negative and positive controls, respectively. After 12 h, a 5 mm diameter disc of *M. yunnanensis* was inoculated at the center of each PDA medium containing different concentrations of T6 peptaibols, sterile water and 1% methanol, respectively. Plates were then incubated in a growth chamber at 25 °C, with each treatment and control group replicated four times. On the fifth day post-inoculation, colonies diameters of *M. yunnanensis* were measured using a cross-measurement method, and the inhibitory rate of T6 peptaibols on *M. yunnanensis* growth was calculated using the specified formula:

Inhibitory rate (%) = (Colony diameter in negative control group − Colony diameter in treatment group)/(Colony diameter in negative control group − 5) × 100

### 2.4. Control Efficacy of T6 Peptaibols on Brown Rot Disease in Pear Fruit

The healthy harvested pear fruits were washed with sterile water, and then disinfected with 75% alcohol, and subsequently thoroughly washed with sterile water three times. Next, they were placed on a sterilized workspace to air dry. A 1250 µg/mL concentration of T6 peptaibols, 1% methanol, and sterile water were sprayed onto the surface of the pear fruits. The treated pears were then left on a sterile workspace for 24 h. After 24 h, a 5 mm diameter and 5 mm deep hole was made at the center of the treated pear fruit, and a *M. yunnanensis* disc was inserted into the hole. The treatment groups included pear fruits treatment with T6 peptaibols (1250 μg/mL) and inoculated with *M. yunnanensis*, with sterile water and inoculated with *M. yunnanensis* (negative control), with 1% methanol and inoculated with *M. yunnanensis* (positive control), and with sterile water, 1% methanol, T6 peptaibols (1250 μg/mL) only. Each treatment and control were repeated three times. The treated pears were then incubated in a climate-controlled chamber at 22 °C for 6 days. After incubation, the pear fruits were photographed and observed. The lesion diameters were measured using the cross-measurement method, and lesion area and inhibitory rate were calculated based on the ellipse area formula. Finally, the pear fruits were cut transversely to measure the depth of decay.

Lesion expansion area (mm^2^) = πab (where a and b represent the length of the major and minor axes of the ellipse, respectively)

### 2.5. Treatment of Test Samples

For the samples collection, 0.1 g of pear fruit flesh from the boundary between healthy and diseased fruits tissue was sampled at different time points (1, 3, 5, 7, and 9 days). For the control group, 0.1 g of healthy fruit flesh was taken at the same time points. The sampled fruit flesh was placed in a sterilized mortar, and 1 mL of CAT, SOD, POD, PPO, PAL, LOX, CHI, and β-Glu enzyme extraction solutions was added, respectively. After thorough grinding, the mixture was collected into a 1.5 mL sterile tube, respectively. The samples were centrifuged at 8000 rpm and 4 °C for 10 mins, and the supernatant was transferred into a sterile tube and kept on ice for further analysis, respectively.

### 2.6. Assessment of Physiological and Biochemical Parameters

At different time intervals (1, 3, 5, 7, 9 days), the contents of malondialdehyde (MDA), hydrogen peroxide (H_2_O_2_) and enzyme activity were measured according to the manufacturer’s protocol using the assay kits (Solarbio, Beijing, China). The absorbance of the MDA sample was measured at three different wavelengths, 450 nm, 532 nm, and 600 nm, and H_2_O_2_ at 415 nm using a spectrophotometer (EPOCH2 Plate Reader, BioTek, Winooski, VT, USA). The contents of MDA and H_2_O_2_ were expressed as μmol/g FW (fresh weight). The antioxidant activities of CAT, SOD, POD, PPO, PAL, LOX, CHI and β-Glu in pear fruit samples were measured according to the manufacturer’s protocol using the assay kits (Solarbio, China). The activities of SOD, POD, PAL and CAT were measured at 560, 470, 290, and 240 nm, respectively, using a spectrophotometer (EPOCH2 Plate Reader, BioTek, Winooski, VT, USA). The experiments were repeated three times.

### 2.7. Measurement of Electrolyte Leakage in Pear fruit

The measurement of electrolyte leakage was performed according to the method described by Cao et al. [22]. The collected pear fruit sample of 0.1 g was placed in a 10 mL sterile tube containing 20 mL of deionized water. After incubation at room temperature for 60 mins, the conductivity (C1) was measured at room temperature using a conductivity meter. The sample was then placed in a boiling water bath for 15 mins to achieve 100% conductivity (C2). Electrolyte leakage was calculated using the formula: Electrolyte Leakage (%) = (C1/C2) × 100.

### 2.8. Analysis of Defense-Related Enzyme Genes Expression by Quantitative Reverse Transcriptase–PCR (qRT–PCR)

The samples for treatment and control groups were collected at 3 days after inoculation, and the samples’ total RNA was extracted using the E.Z.N.A.^®^ Plant RNA Kit (Tiangen Biotechnology, Beijing, China). The quantity and quality of the total RNA were assessed using a NanoDrop spectrophotometer at absorbance wavelengths of 230 and 260 nm. First-strand cDNA synthesis was performed using the RevertAid™ First Strand cDNA Synthesis Kit (Tiangen Biotechnology, Beijing, China). The final concentrations of total RNA in treatment and control groups were adjusted to be the same using RNase-free water. The specific primers for the target genes of the defense-related enzymes (*CAT*, *SOD*, *POD*, *PPO*, *PAL*, *LOX*, *CHI*, and *β-Glu*) and the internal control actin gene (Table 2) were designed based on the EST sequences available in NCBI by using Primer Express 5.0 software, and were used to amplify genes-specific amplicons. The qRT–PCR was conducted in a 20 μL reaction volume using Heff SYBR Green Master Mix, with 1 μL of cDNA solution and 10 μM primers. The relative expression levels of defense-related enzyme genes were determined using the 2^−ΔΔCt^ method described by Livak and Schmittgen [23].

### 2.9. Data Analysis

The T6 peptaibols concentration was selected based on its inhibitory rate on *M. yunnanensis*. The data in the present study were mean ± standard error, analyzed by ANOVA, and statistical analysis of the data was performed using MS Office Excel 2021 and IBM SPSS Statistics 27 software. Multiple comparisons were conducted using Duncan’s new multiple range test and significance was considered at *p* < 0.05.

## 3. Results

### 3.1. Antifungal Activity of T6 Peptaibols on M. yunnanensis and Disease Control

The T6 peptaibols with different concentrations ranging from 250 to 1250 µg/mL had significant inhibitory effect on the colony growth of *M. yunnanensis* (Table 3). An increase in T6 peptaibols concentration increases the inhibitory rate significantly. The highest inhibitory rate was recorded at 1250 µg/mL with 85.99% compared to the negative control (sterile water) at 5 days after treatment with T6 peptaibols, whereas the lowest inhibitory rate of 35.79% was recorded at 5 days with a concentration of 250 µg/mL in comparison to the negative control. In contrast, the colony diameter of the positive control (1% methanol) was significantly bigger than that of the treatment group, whereas it was slightly smaller than that of the negative control. Compared with the negative control, the average colony diameter of *M. yunnanensis* in the positive control group was reduced by only 5.56 mm, with an inhibition rate of 8.72%. Therefore, the concentration of 1250 µg/mL was selected for further experiment.

### 3.2. Controlling Effect of T6 Peptaibols on Brown Rot Disease in Pear Fruit

**Figure 1:**
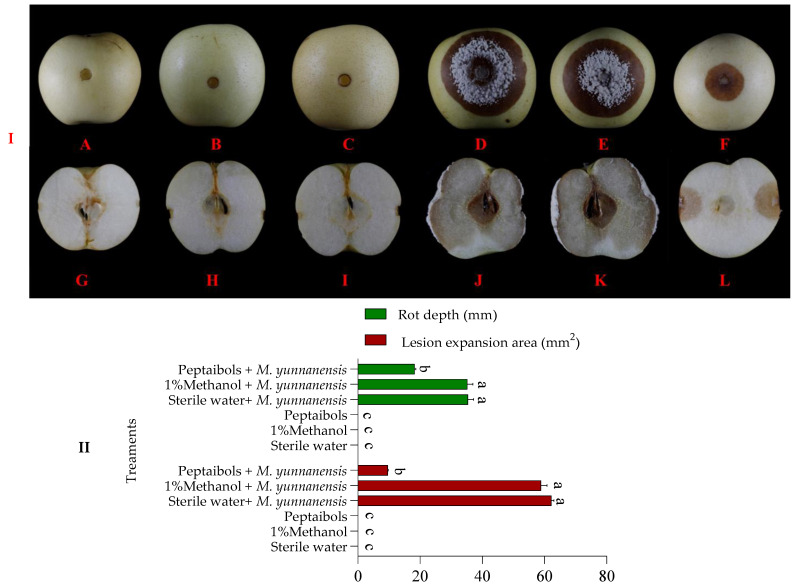
(**I**) Effect of T6 peptaibols on the expansional symptoms of brown rot lesions on pear fruits. (**A**) the lesion of healthy pear fruit treated with sterile water only; (**B**) the lesion of healthy pear fruit treated with 1% methanol only, (**C**) the lesion of healthy pear fruit treated with T6 peptaibols only, (**D**) the lesion of pear fruit treated with *M. yunnanensis* and sterile water (negative control), (**E**) the lesion of pear fruit treated with 1% methanol and *M. yunnanensis* (positive control), (**F**) the lesion of pear fruit treated with T6 peptaibols and *M. yunnanensis*, (**G**) cross-section of healthy pear fruit treated with water only, (**H**) cross-section of pear fruit treated with 1% methanol only, (**I**) cross-section of pear fruit treated with T6 peptaibols only, (**J**) cross-section of pear fruit treated with *M. yunnanensis* and sterile water (negative control), (**K**) cross-section of pear fruit treated with 1% methanol and *M. yunnanensis* (positive control), and (**L**) cross-section of pear fruit treated with T6 peptaibols and *M. yunnanensis*. (**II**) Effect of 1250 µg/mL concentration of T6 peptaibols on lesions expansion in pear fruits after inoculation with *M. yunnanensis*; Small bars represent the standard errors of the means. Different lowercase letters indicate significant differences at *p* < 0.05.

In the in vitro setup, the T6 peptaibols significantly inhibited the lesion expansion on pear fruit with brown rot at 6 days after inoculation. The size of the rot lesions on pear fruit was significantly smaller than the negative (sterile water and *M. yunnanensis*) (Figure 1(ID)) and positive controls (1% methanol and *M. yunnanensis*) (Figure 1(IE)) at 6 days after treatment with T6 peptaibols and inoculation with *M. yunnanensis* (Figure 1(IF)), whereas the center of the rot lesions with abundant mycelium in the negative (Figure 1(ID)) and positive (Figure 1(IE)) pear fruits, and the rot lesion size on the negative fruit was larger than that of positive fruit, but with no significant difference. Finally, no lesion expansion and mycelium were observed on the surface of healthy pear fruits treated with sterile water, 1% methanol and T6 peptaibols only (Figure 1(IA–C)). In addition, the lesion area on pear fruit was 9.58 mm² after treatment with T6 peptaibols and *M. yunnanensis*, whereas the lesions area of 62.16 mm² and 58.88 mm² were recorded on negative and positive pear fruits, respectively. The inhibitory rate of the lesion area was 84.57% at 6 days after inoculation with T6 peptaibols and *M. yunnanensis* in comparison to the negative control (Figure 1II).

From a cross-sectional view, pear fruits in negative and positive control groups exhibited a significant brown cross-section with complete core decay (Figure 1(IJ,K)), and the decay depths were 35.40 mm and 35.20 mm (Figure 1II), respectively. In contrast, pear fruit treated with T6 peptaibols and inoculated with *M. yunnanensis* showed a smaller brown cross-section with no core decay (Figure 1(IL)), the decay depth was only 18.30 mm (Figure 1II), and the depth reduction rate was 48.31% in comparison to the negative control. However, the healthy pear fruits treated with sterile water, 1% methanol and T6 peptaibols only showed no lesion expansion (Figure 1(IG–I,II)).

### 3.3. Effects of T6 Peptaibols on Oxidative Alleviation and Electrolyte Leakage for Pear Fruit

**Figure 2:**
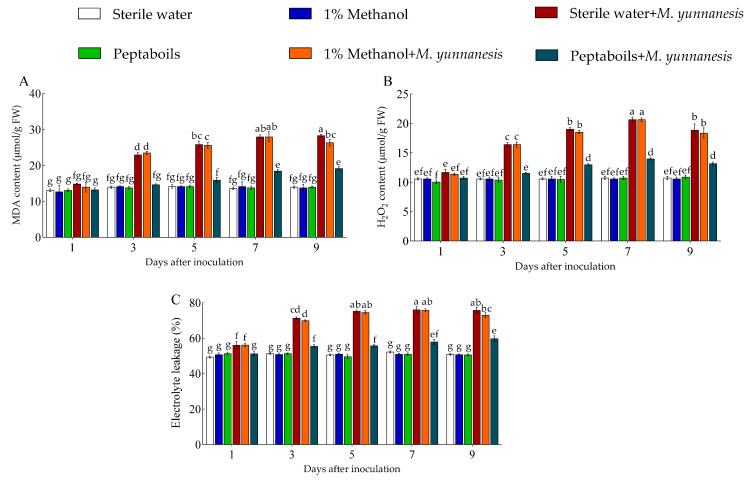
Effect of T6 peptaibols on (**A**) MDA content, (**B**) H_2_O_2_ content and (**C**) Electrolyte leakage of pear fruits at different days. Small bars represent the standard errors of the means. Different lowercase letters indicate significant differences at *p* < 0.05.

The MDA content (Figure 2A), H_2_O_2_ content (Figure 2B), and electrolyte leakage (Figure 2C) in pear fruits treated with T6 peptaibols and inoculated with *M. yunnanensis* gradually increased, and then remained stable or slightly decreased. From days 1 to 9 after inoculation, the average values of MDA content, H_2_O_2_ content, and electrolyte leakage in healthy pear fruit treated with sterile water only were 13.76 μmol/g, 10.63 μmol/g, and 50.89%, respectively, whereas 13.80 μmol/g, 10.57 μmol/g, and 50.86% were recorded in the pear fruit treated with 1% methanol only. However, pear fruit treated with sterile water and inoculated with *M. yunnanensis* exhibited the average MDA content, H_2_O_2_ content, and electrolyte leakage values of 23.98 μmol/g, 17.33 μmol/g, and 70.91%, respectively, while the average values of 16.31 μmol/g, 12.49 μmol/g, and 56.02% were recorded in the pear fruit treated with T6 peptaibols and inoculated with *M. yunnanensis*, and decreased by 31.99%, 27.93%, and 21.00% in comparison to the pear fruit treated with sterile water and inoculated with *M. yunnanensis*, respectively. However, compared to 1% methanol and *M. yunnanensis* there was no significance difference in the fruit treated with sterile water and *M. yunnanensis* (MDA content, H_2_O_2_ content, and electrolyte leakage). Additionally, compared to the pear fruit treated with T6 peptaibols only, the average MDA content, H_2_O_2_ content, and electrolyte leakage increased by 20.36%, 18.16%, and 10.15% after treatment with T6 peptaibols and inoculation with *M. yunnanensis* ranging from days 1 to 9, respectively.

### 3.4. Effect of T6 Peptaibols on Defense Enzyme Activity

**Figure 3:**
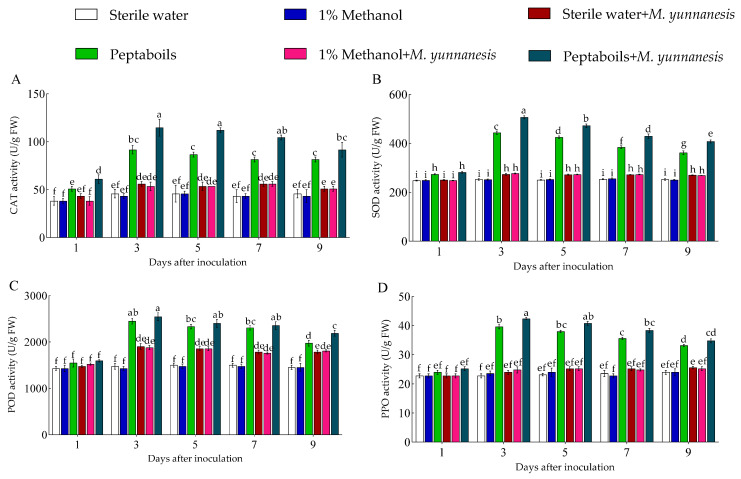
Effect of T6 peptaibols treatment on (**A**) CAT, (**B**) SOD, (**C**) POD and (**D**) PPO antioxidant enzyme activities of pear fruits at different days. Small bars represent the standard errors of the means. Different lowercase letters indicate significant differences at *p* < 0.05.

The CAT, SOD, POD, and PPO enzyme activities of pear fruits treated with T6 peptaibols and inoculation with *M. yunnanensis* were significantly higher than other treatment groups at different time points (1, 3, 5, 7, and 9 days), showing a trend of a gradual increase followed by a decrease. From days 1 to 9, the average enzyme activities of CAT (Figure 3A), SOD (Figure 3B), POD (Figure 3C), and PPO (Figure 3D) in healthy pear fruits treated with sterile water only were 43.83, 251.69, 1469.40, and 23.28 U/g, respectively. In healthy pear fruits treated with 1% methanol only, the average enzyme activities of CAT, SOD, POD, and PPO were 42.81, 252.07, 1450.38, and 23.44 U/g, showing no significant differences compared to those treated with sterile water. However, in healthy pear fruits treated with T6 peptaibols, these enzymes exhibited average activities of 78.49, 377.74, 2120.88, and 34.08 U/g, respectively, representing increases of 79.07%, 50.08%, 44.34%, and 46.39% compared to healthy fruits treated with sterile water only. Furthermore, in pear fruits treated with sterile water and inoculated with *M. yunnanensis*, the average enzyme activities of CAT, SOD, POD, and PPO were 51.97, 267.67, 1759.47, and 24.56 U/g, respectively, and 96.84, 419.62, 2215.98, and 36.32 U/g after treatment with T6 peptaibols and inoculation with *M. yunnanensis*, and the average enzyme activities increased by 86.27%, 56.76%, 25.94%, and 47.88% in comparison to the pear fruits treated with sterile water and inoculated with *M. yunnanensis*, respectively. However, compared to 1% methanol and *M. yunnanensis*, there was no significance difference in the fruits treated with sterile water and *M. yunnanensis* antioxidant enzymes activity. Compared to healthy pear fruits treated with T6 peptaibols only, the average enzyme activity of CAT, SOD, POD, and PPO in fruits treated with T6 peptaibols and inoculated with *M. yunnanensis* increased by 23.38%, 11.09%, 4.48%, and 6.57%, respectively.

### 3.5. Effect of T6 Peptaibols on Enzyme Activities in Metabolic Pathway

**Figure 4:**
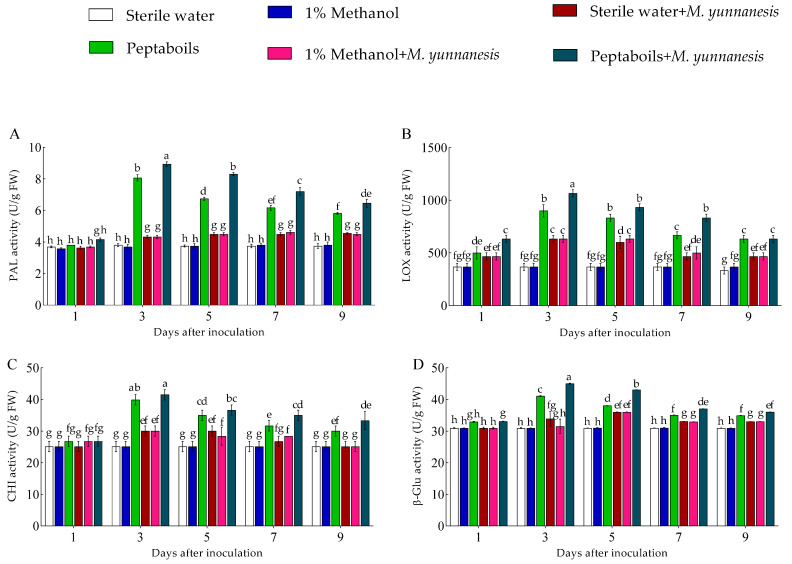
Effect of T6 peptaibols treatment on (**A**) PAL, (**B**) LOX, (**C**) CHI and (**D**) β-Glu antioxidant enzyme activities of pear fruits at different days. Small bars represent the standard errors of the means. Different lowercase letters indicate significant differences at *p* < 0.05.

The activities of PAL, LOX, CHI, and β-Glu enzymes in pear fruits treated with T6 peptaibols were significantly higher than other treatment groups at different time points (1, 3, 5, 7, and 9 days), showing a trend of first increasing and then gradually decreasing. During the 1–9 days post-treatment, the average enzyme activities of PAL (Figure 4A), LOX (Figure 4B), CHI (Figure 4C), and β-Glu (Figure 4D) in healthy pear fruits treated with sterile water only were 3.75, 360, 25.16, and 30.99 U/g, respectively. For healthy pear fruits treated with 1% methanol only, the average enzyme activities of PAL, LOX, CHI, and β-Glu were 3.73, 366.67, 25.16, and 31 U/g, and showing no significant differences compared to those treated with sterile water only. However, in healthy pear fruits treated with T6 peptaibols only, the average enzyme activities of PAL, LOX, CHI, and β-Glu were 6.12, 706.67, 32.68, and 36.39 U/g, representing increases of 63.38%, 92.29%, 29.87%, and 17.43%, respectively, compared to the sterile water-treated group. For pear fruits treated with sterile water and inoculated with *M. yunnanensis*, the average enzyme activities of PAL, LOX, CHI, and β-Glu were 4.30, 526.67, 27.45, and 33.67 U/g, respectively. However, compared to 1% methanol and *M. yunnanensis*, there was no significance difference in the fruits treated with sterile water and *M. yunnanensis*. In contrast, pear fruits treated with T6 peptaibols and inoculated with *M. yunnanensis*, the average enzyme activities were 7.01, 820.00, 34.64, and 38.82 U/g, showing increases of 63.00%, 55.70%, 26.19%, and 16.34%, respectively. In addition, compared to healthy pear fruits treated with T6 peptaibols only, the average enzyme activities of PAL, LOX, CHI, and β-Glu in fruit treated with T6 peptaibols and inoculated with *M. yunnanensis* were increased by 14.54%, 16.04%, 5.99%, and 6.68%, respectively.

### 3.6. Effect of T6 Peptaibols on the Expression Levels of Defense-Related Genes in Pear Fruit

**Figure 5:**
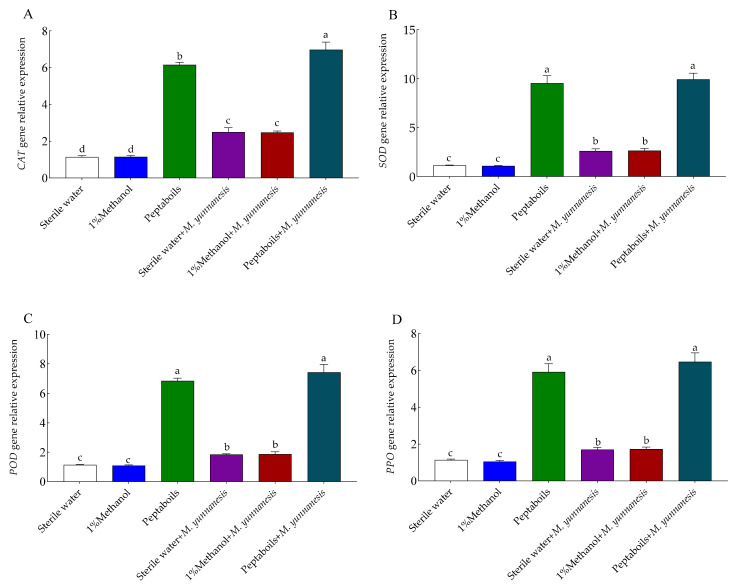
Effect of T6 peptaibols on the relative expression levels of (**A**) *CAT*, (**B**) *SOD*, (**C**) *POD* and (**D**) *PPO* antioxidants genes of pear fruits at day 3. Small bars represent the standard errors of the means. Different lowercase letters indicate significant differences at *p* < 0.05.

After treatment with T6 peptaibols, the expression levels of defense-related genes in pear fruits were significantly increased. On day 3, the expression levels of *CAT* (Figure 5A), *SOD* (Figure 5B), *POD* (Figure 5C), and *PPO* (Figure 5D) genes in healthy pear fruits treated with sterile water or 1% methanol showed no significant differences. However, in healthy pear fruits treated with T6 peptaibols only, the expression levels of *CAT*, *SOD*, *POD*, and *PPO* genes increased by 4.41, 7.38, 5.03, and 4.21-folds, respectively, compared to those in sterile water-treated healthy pear fruits. In pear fruits treated with sterile water and inoculated with *M. yunnanensis*, the expression levels of *CAT*, *SOD*, *POD*, and *PPO* genes increased by 1.19, 1.30, 0.62, and 0.50-folds, respectively, compared to those in sterile water-treated healthy pear fruits. Furthermore, in pear fruits treated with T6 peptaibols and inoculated with *M. yunnanensis*, the expression levels of *CAT*, *SOD*, *POD*, and *PPO* genes increased by 2.80, 2.81, 3.03, and 2.79-folds, respectively, compared to those in sterile water-treated pear fruits and inoculated with *M. yunnanensis*. However, compared to 1% methanol and *M. yunnanensis*, there was no significance difference in the fruits treated with sterile water and *M. yunnanensis* antioxidant genes expressions. Compared to healthy pear fruits treated with T6 peptaibols, the expression levels of *CAT*, *SOD*, *POD*, and *PPO* genes in pear fruits treated with T6 peptaibols and inoculated with *M. yunnanensis* further increased by 0.13, 0.04, 0.08, and 0.09-folds, respectively.

**Figure 6:**
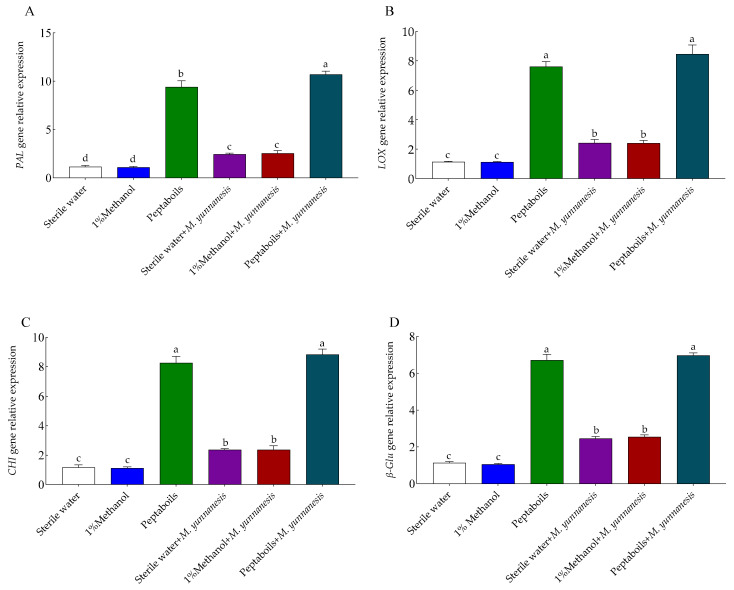
Effect of T6 peptaibols on the relative expression levels of (**A**) *PAL*, (**B**) *LOX*, (**C**) *CHI* and (**D**) *β-Glu* defense related genes of pear fruits at day 3. Small bars represent the standard errors of the means. Different lowercase letters indicate significant differences at *p* < 0.05.

Furthermore, on day 3 the expression levels of the *PAL* (Figure 6A), *LOX* (Figure 6B), *CHI* (Figure 6C), and *β-Glu* (Figure 6D) defense-related genes in healthy pear fruits treated with sterile water or 1% methanol showed no significant differences. In contrast, in healthy pear fruits treated with T6 peptaibols only, the expression levels of these genes increased by 7.20, 5.70, 6.11, and 4.90-folds, respectively, compared to those in sterile water-treated healthy pear fruits. In pear fruits treated with sterile water and inoculated with *M. yunnanensis*, the expression levels of *PAL*, *LOX*, *CHI*, *and β-Glu* genes increased by 1.13, 1.14, 1.04, and 1.16-folds, respectively, compared to those in sterile water-treated healthy pear fruits. However, there was no significance difference in the fruits treated with sterile water and *M. yunnanensis* genes expression, compared to 1% methanol and *M. yunnanensis* treated fruits. In contrast, pear fruits treated with T6 peptaibols and inoculated with *M. yunnanensis*, the expression levels of these genes increased by 3.37, 2.49, 2.73, and 1.83-folds, respectively, compared to those in sterile water-treated pear fruit inoculated with *M. yunnanensis*. Compared to healthy pear fruits treated with T6 peptaibols, the expression levels of the *PAL*, *LOX*, *CHI*, and *β-Glu* genes in pear fruits treated with T6 peptaibols and inoculated with *M. yunnanensis* further increased by 0.14, 0.11, 0.07, and 0.04-folds, respectively.

## 4. Discussion

The sustained attention given to novel peptaibols derived from fungi is primarily attributed to their diverse biological activities, including antibacterial, hemolytic, and antifungal properties. Our study demonstrated that peptaibols produced by *Trichoderma longibrachiatum* T6 (T6) exhibit inhibitory effects against *M. yunnanensis*. A previous study reported that the antimicrobial peptide Trichokonin VI (TK VI), isolated from *T. pseudokoningii* SMF2, inhibited the growth of *B. cinerea* by suppressing mycelial growth. The inhibitory effect increased with the concentration of TK VI, reaching an inhibitory rate of 90.6% at 160 mg/L [24]. Similarly, peptaibol extracts (Paib) from *T. asperellum* inhibited the growth of pathogens *Colletotrichum gloeosporioides*, *B. cinerea*, *A. alternata*, and *F. oxysporum* by 92.20%, 74.20%, 58.40%, and 36.20%, respectively, at a concentration of 800 μg/mL [25]. Our results showed that peptaibols produced by T6 also exhibit strong inhibitory activity against *M. yunnanensis*, achieving an inhibitory rate of 85.99% at 1250 μg/mL.

The inhibition efficiency of T6 peptaibols on pear brown rot lesion expansion reached 84.67%. In consistent with previous results, Pamela et al. [25] reported that tomatoes treated with peptaibols extracted from *T. asperellum* had an infection rate of 0%, while untreated tomatoes showed an infection rate of 92.5%. Similarly, Zhao et al. [24] found that the antimicrobial peptide Trichokonins (TKs) extracted from *T. pseudokoningii* SMF2 at 200 mg/L achieved an inhibition rate of 76.70% against gray mold after treatment for moth orchids.

ROS have a significant role in plant response to pathogenic stress, but the pattern varies between necrotrophs and biotrophs [26,27,28,29]. A finetuned systemic induction system is involved in ROS-mediated disease development in plants. In regulated concentrations, ROS act as a signaling molecule and activate different pathways to suppress pathogens. However, an excess of these ROS is deleterious to plant systems [30]. In current study, we found that T6 peptaibols significantly decreased the contents of MDA and H_2_O_2_, as well as electrolyte leakage of pear fruits after inoculation with *M. yunnanensis*, compared to the sterile water + *M. yunnanensis* treated fruits. Excessive ROS discharge in plants while interacting with a pathogen can strike both partners. However, plants protect themselves by maintaining a perfect balance between ROS synthesis and ROS-scavenging mechanisms. Plants accomplish ROS equilibrium by enzymatic or non-enzymatic antioxidant defense mechanisms that tightly compartmentalize the regulation of ROS levels [31,32,33,34]. The decrease in brown rot lesion on the T6-treated pear fruits and low accumulation of ROS were due to the increased enzymatic or non-enzymatic antioxidant defense mechanisms in the current study. The antioxidant enzyme activity of pear fruits treated with T6 peptaibols increased across the different number of days after inoculation with *M. yunnanensis* compared to the controls, respectively. Compared to the controls, T6 peptaibols increased SOD, POD, CAT and PPO activity across the different days. Likewise, the combined *M. yunnanensis* + T6 peptaibols treated samples increased SOD, POD, CAT and PPO activities significantly compared to sterile water + *M. yunnanensis* treated samples. In addition, the enzyme activity of PAL, a key enzyme in the phenylpropanoid metabolic pathway, as well as other defense-related enzymes such as LOX, β-Glu, and CHI, were significantly increased after treatment with T6 peptaibols + *M. yunnanensis*. Compared to previous study, the application of metabolite Trichokonins from *T. pseudokoningii* SMF2 significantly increased the production of phenolic compounds, as well as the activity of enzymes such as PAL, POD and PPO in tobacco, indicating that resistance to TMV infection in tobacco was enhanced through the application of Trichokonins [18]. Application of alamethicin, a long sequence peptaibol with a 20-residue produced by *T. viride*, induced defense responses in *Phaseolus lunatus* [35] and *Arabidopsis thaliana* [36]. These findings are consistent with previous findings [37], which reported that some metabolites have low molecular weights and play a significant role in non-enzymatic antioxidant defense, firmly relating the cellular antioxidant capability to the preservation of redox equilibrium.

In the current study, compared to the negative control, T6 peptaibols and the combined *M. yunnanensis* + T6 peptaibols treated samples increased the antioxidant and defense related genes transcript levels, respectively. The combined *M. yunnanensis* + T6 peptaibols treatment increased the *CAT*, *SOD*, *POD*, and *PPO* antioxidant genes expression. In addition, the combined *M. yunnanensis* + T6 peptaibols treatment increased the *CHI*, *LOX*, *PAL* and *β-Glu* defense related genes expression, respectively, compared to negative control. The increase in both antioxidant and defense related genes expression stimulated by the T6 peptaibols to decrease the *M. yunnanensis* infection on pear fruit. Luo et al. [18] reported that the application of Trichokonins, metabolites from *T. pseudokoningii* SMF2, in tobacco upregulated the expression of defense-related genes *SOD*, *CAT*, *APX*, and *POX* by 1.80, 1.80, 2.50, and 2.30-folds, respectively, demonstrating that Trichokonins mediated resistance to TMV by activating multiple defense pathways in tobacco. Overall, our findings suggest that T6 peptaibols could be a promising biocontrol agent for pear brown rot, necessitating further research to clarify these mechanisms and optimize application strategies. However, the inhibitory effects of T6 peptaibols on other plant pathogens’ growth and disease control, and the molecular mechanisms for its inducing plant resistance, will be further studied in the future.

## 5. Conclusions

In this study, T6 peptaibols were effective in inhibiting the growth of *M. yunnanensis* and controlling brown rot disease. The treatment of pear fruits stimulated immunity against *M. yunnanensis* by suppressing the pathogen growth, increasing antioxidants, promoting phenylpropanoid metabolic pathways, and reducing ROS accumulation. Our findings in this current study provided the underlying strategies and pathway of T6 peptaibols in controlling brown rot in pear fruit.

## Figures and Tables

**Table 1:** XAD-2 macro-porous resin elution gradients.

Fractions Volume (mL)	Elution Gradients	Flow Rate (mL/min)
700	V_(Water)_:V_(Methanol)_ = 100:0	1
700	V_(Water)_:V_(Methanol)_ = 40:60	1
700	V_(Water)_:V_(Methanol)_ = 0:100	1

**Table 2:** qRT–PCR primers for determining defense-related enzyme genes expression.

Genes Name	Primers Sequence (5′-3′)	Genes ID
*CAT*	CGTGGCAGCCCTGAAAC	XR_007254874.1
	ATTCTCTTGGATGTGGGACTTA	
*SOD*	TTCTTGGGCGAGCAGTTG	XM_009354853.3
	TCCTGCGTTCCCAGTAGTCT	
*POD*	TCAGGCAGAAGTTTGAGCAG	XM_009369735
	CGCTCTCAAGACCAAGGACA	
*PPO*	CTCAGCAGGCTACAAGGCA	XM_048591029.1
	GACATTTACAACCGCATCACT	
*PAL*	ACGCAAAATGGTCAAAACG	XM_009375248.3
	AGACTCTCTCCGCCGAGC	
*LOX*	ATCCATTCCGAACGACCA	XM_048566477
	TCTCCCCAGTTCCATCTCC	
*CHI*	CCCTCTTCACTCATCTTTTCTG	XM_009360218.3
	CAAGGATAGCACCGCCAC	
*β-Glu*	GAAGATAAGGACATTTTCGGG	XM_009355010.3
	CTGATAAAGGAGAGCCACTACG	
*Actin*	TGCTGAGCGGGAAATTGTG	AF386514.1
	TCCGATAGTGATTACCTGTCCATC	

**Table 3:** Effect of different concentrations of T6 peptaibols on the colony diameters of *M. yunnanensis*.

Concentrations (µg/mL)	Colony Diameters (mm)	Inhibitory Rates (%)
250	45.94 ± 0.61 c	35.79 ± 0.96 e
500	29.97 ± 0.22 d	60.83 ± 0.34 d
750	22.07 ± 0.35 e	73.23 ± 0.54 c
1000	17.17 ± 0.38 f	80.91 ± 0.59 b
1250	13.94 ± 0.14 g	85.99 ± 0.22 a
Positive control	63.20 ± 0.28 b	8.72 ± 0.44 f
Negative control	68.76 ± 0.82 a	_

The positive control group was treated with a solution containing 1% methanol, while the negative control was treated with sterile water. Data presented are mean ± SE for four replicates. Different letters within each column indicate significant difference among treatments at *p* < 0.05 level.

## Data Availability

The data presented in this study are available on request from the corresponding authors.
